# 4-[1-(4-Cyano­benz­yl)-1*H*-benzimidazol-2-yl]benzonitrile

**DOI:** 10.1107/S1600536809006989

**Published:** 2009-03-06

**Authors:** Reza Kia, Hoong-Kun Fun, Hadi Kargar

**Affiliations:** a X-ray Crystallography Unit, School of Physics, Universiti Sains Malaysia, 11800 USM, Penang, Malaysia; b Department of Chemistry, School of Science, Payame Noor University (PNU), Ardakan, Yazd, Iran

## Abstract

In the title compound, C_22_H_14_N_4_, a new substituted benzimidazole, three inter­molecular C—H⋯N inter­actions link neighbouring mol­ecules into different dimers with *R*_2_^2^(12), *R*_2_^2^(8) and *R*_2_^2^(24) ring motifs. A fourth C—H⋯N inter­action links neighbouring mol­ecules along the *c* axis. There is also a short inter­molecular contact between the azomethine (C=N) segment of the benzimidazole ring and one of the C atoms of a neighbouring benzene ring [N⋯C = 3.191 (5), C⋯C = 3.364 (6) Å], which links the mol­ecules along the *a* axis. The two cyano­benzene rings are almost perpendicular to each other, with an inter­planar angle of 87.70 (7)°. The dihedral angles between the mean planes of the benzimidazole ring and the two outer benzene rings are 36.27 (16) and 86.70 (16)°. In the crystal structure, mol­ecules are stacked down the *a* axis with centroid–centroid distances of 3.906 (2)–3.912 (2) Å and inter­planar distances of 3.5040 (17) and 3.6235 (17) Å.

## Related literature

For hydrogen-bond motifs, see: Bernstein *et al.* (1995 ▶). For benzimidazole chemistry, reaction mechanisms and their bioactivity, see, for example: Latif *et al.* (1983 ▶); Craigo *et al.* (1999 ▶); Gudmundsson *et al.* (2000 ▶); Trivedi *et al.* (2006); Kim *et al.* (1996 ▶); Ramla *et al.* (2006 ▶). For the stability of the temperature controller used in the data collection, see: Cosier & Glazer (1986 ▶).
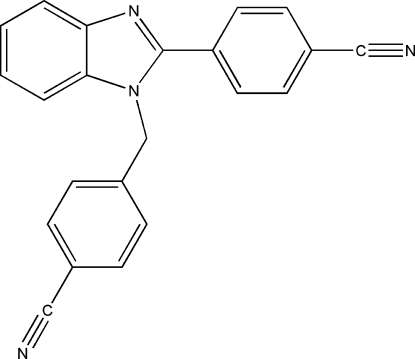


## Experimental

### 

#### Crystal data

C_22_H_14_N_4_
*M* *_r_* = 334.37
Monoclinic, 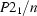

*a* = 5.0553 (4) Å
*b* = 17.3437 (10) Å
*c* = 19.6339 (14) Å
β = 97.653 (5)°
*V* = 1706.1 (2) Å^3^
*Z* = 4
Mo *K*α radiation
μ = 0.08 mm^−1^
*T* = 100 K
0.45 × 0.09 × 0.04 mm

#### Data collection

Bruker SMART APEXII CCD area-detector diffractometer
Absorption correction: multi-scan (*SADABS*; Bruker, 2005 ▶) *T*_min_ = 0.965, *T*_max_ = 0.997
15641 measured reflections
2906 independent reflections
1490 reflections with *I* > 2σ(*I*)
*R*_int_ = 0.153

#### Refinement

*R*[*F* ^2^ > 2σ(*F* ^2^)] = 0.079
*wR*(*F* ^2^) = 0.169
*S* = 1.07
2906 reflections
236 parameters
H-atom parameters constrained
Δρ_max_ = 0.26 e Å^−3^
Δρ_min_ = −0.35 e Å^−3^

### 

Data collection: *APEX2* (Bruker, 2005 ▶); cell refinement: *SAINT* (Bruker, 2005 ▶); data reduction: *SAINT*; program(s) used to solve structure: *SHELXTL* (Sheldrick, 2008 ▶); program(s) used to refine structure: *SHELXTL*; molecular graphics: *SHELXTL*; software used to prepare material for publication: *SHELXTL* and *PLATON* (Spek, 2009 ▶).

## Supplementary Material

Crystal structure: contains datablocks global, I. DOI: 10.1107/S1600536809006989/zl2173sup1.cif

Structure factors: contains datablocks I. DOI: 10.1107/S1600536809006989/zl2173Isup2.hkl

Additional supplementary materials:  crystallographic information; 3D view; checkCIF report

## Figures and Tables

**Table 1:** Hydrogen-bond geometry (Å, °)

*D*—H⋯*A*	*D*—H	H⋯*A*	*D*⋯*A*	*D*—H⋯*A*
C12—H12*A*⋯N1^i^	0.93	2.62	3.425 (5)	146
C19—H19*A*⋯N4^ii^	0.93	2.57	3.502 (6)	175
C9—H9*A*⋯N4^iii^	0.93	2.71	3.361 (5)	128
C14—H14*A*⋯N3^iv^	0.97	2.57	3.502 (5)	162

Symmetry codes: (i) 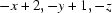; (ii) 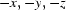; (iii) 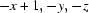; (iv) 
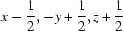
.

**Fig. 1.**
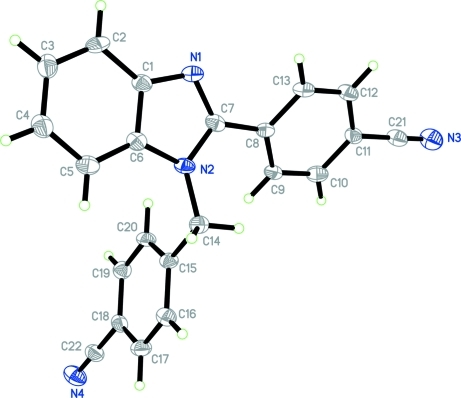
The molecular structure of the title compound with atom labels and 50% probability ellipsoids for non-H atoms.

**Fig. 2.**
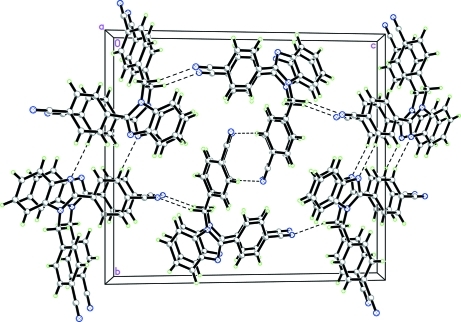
The crystal packing of the title compound, viewed down the a-axis showing infinite stacks of molecules along the a-axis and also linking of molecules through C—H···N interactions along the c-axis. Intermolecular hydrogen bonds are shown as dashed lines.

## supplementary crystallographic information

### Comment

Benzimidazoles are used widely in biological applications and as pharmaceutical agents (Craigo *et al.*, 1999; Gudmundsson *et al.*, 2000; Trivedi *et al.*, 2006). They are also used as topoisomerase I inhibitors (Kim *et al.*, 1996) and for antitumor activity (Ramla *et al.*, 2006). Due to these important applications, many synthetic routes towards benzimidazoles have been developed. They can, for example, be synthesized by the reaction of phenolic aldehydes with *o*-phenylenediamine (Latif *et al.*, 1983). Based on this route the title compound was synthesized and its crystal structure is reported here.

The title compound, Fig.1, comprises a single molecule in the asymmetric unit. Three intermolecular C—H···N interactions link neighbouring molecules into different dimers with *R*_2_^2^(12), *R*_2_^2^(8) and *R*_2_^2^(24) ring motifs (Bernstein *et al.*, 1995). A fourth C—H···N interaction links neighbouring molecules along the *c* axis. The two cyanobenzene rings are almost perpendicular to each other, with an interplanar angle of 87.70 (7)°. The dihedral angles between the the mean planes of the benzimidazole ring and the two outer benzene rings are 36.27 (16) and 86.70 (16)°. There is also a short intermolecular contact between the azomethine (C1═N1) segment of the benzimidazole ring and one of the carbon atoms (C13) of the neighbouring benzene rings which links the molecules along the *a* axis (N1···C13^v^ = 3.191 (5) C1···C13^v^ = 3.364 (6), symmetry code: (v) x-1, y, z). In the crystal structure, the molecules are stacked down the *a* axis with centroid to centroid distances of 3.906 (2)–3.912 (2) Å and interplanar distances of 3.5040 (17) and 3.6235 (17) Å [*Cg*1···*Cg*2^vi^ = 3.906 (2); (vi) 1 + *x*, *y*, *z* and *Cg*1···*Cg*3^vii^ = 3.912 (2) Å; (vii) -1 + *x*, *y*, *z*: *Cg*1, *Cg*2 and *Cg*3 are the centroids of the N1/C1/C6/N2/C7, C1–C6, and C8–C13 benzene rings] (Fig. 2).

### Experimental

An ethanolic solution (50 ml) of 4-cyanobenzaldehyde (2 mmol, 263 mg) was added to 1,2-phenylenediamine (1 mmol, 217 mg). The mixture was refluxed for 2 h, and cooled to room temperature. The resulting colourless powder was filtered, washed with cooled ethanol and dried in *vacuo*. Single crystals suitable for *X*-ray diffraction were obtained from an ethanol solution at room temperature.

### Refinement

All hydrogen atoms were positioned gemetrically and refined in a riding model approximation with C—H = 0.93–0.97 Å and *U*_iso_ (H) = 1.2 *U*_eq_ (C).

### Crystal data

**Table:**

C_22_H_14_N_4_	*F*(000) = 696
*M**_r_* = 334.37	*D*_x_ = 1.302 Mg m^−^^3^
Monoclinic, *P*2_1_/*n*	Mo *K*α radiation, λ = 0.71073 Å
Hall symbol: -P 2yn	Cell parameters from 3931 reflections
*a* = 5.0553 (4) Å	θ = 2.4–30.2°
*b* = 17.3437 (10) Å	µ = 0.08 mm^−^^1^
*c* = 19.6339 (14) Å	*T* = 100 K
β = 97.653 (5)°	Needle, colourless
*V* = 1706.1 (2) Å^3^	0.45 × 0.09 × 0.04 mm
*Z* = 4	

### Data collection

**Table:**

Bruker SMART APEXII CCD area-detector diffractometer	2906 independent reflections
Radiation source: fine-focus sealed tube	1490 reflections with *I* > 2σ(*I*)
graphite	*R*_int_ = 0.153
φ and ω scans	θ_max_ = 25.0°, θ_min_ = 1.6°
Absorption correction: multi-scan (*SADABS*; Bruker, 2005)	*h* = −6→6
*T*_min_ = 0.965, *T*_max_ = 0.997	*k* = −20→20
15641 measured reflections	*l* = −23→22

### Refinement

**Table:**

Refinement on *F*^2^	Secondary atom site location: difference Fourier map
Least-squares matrix: full	Hydrogen site location: inferred from neighbouring sites
*R*[*F*^2^ > 2σ(*F*^2^)] = 0.079	H-atom parameters constrained
*wR*(*F*^2^) = 0.169	*w* = 1/[σ^2^(*F*_o_^2^) + (0.0473*P*)^2^ + 1.9898*P*] where *P* = (*F*_o_^2^ + 2*F*_c_^2^)/3
*S* = 1.07	(Δ/σ)_max_ < 0.001
2906 reflections	Δρ_max_ = 0.26 e Å^−^^3^
236 parameters	Δρ_min_ = −0.35 e Å^−^^3^
0 restraints	Extinction correction: *SHELXTL* (Sheldrick, 2008), Fc^*^=kFc[1+0.001xFc^2^λ^3^/sin(2θ)]^-1/4^
Primary atom site location: structure-invariant direct methods	Extinction coefficient: 0.0124 (18)

### Special details

**Table:**

Experimental. The crystal was placed in the cold stream of an Oxford Cyrosystems Cobra open-flow nitrogen cryostat (Cosier & Glazer, 1986) operating at 100.0 (1) K.
Geometry. All e.s.d.'s (except the e.s.d. in the dihedral angle between two l.s. planes) are estimated using the full covariance matrix. The cell e.s.d.'s are taken into account individually in the estimation of e.s.d.'s in distances, angles and torsion angles; correlations between e.s.d.'s in cell parameters are only used when they are defined by crystal symmetry. An approximate (isotropic) treatment of cell e.s.d.'s is used for estimating e.s.d.'s involving l.s. planes.
Refinement. Refinement of *F*^2^ against ALL reflections. The weighted *R*-factor *wR* and goodness of fit *S* are based on *F*^2^, conventional *R*-factors *R* are based on *F*, with *F* set to zero for negative *F*^2^. The threshold expression of *F*^2^ > σ(*F*^2^) is used only for calculating *R*-factors(gt) *etc*. and is not relevant to the choice of reflections for refinement. *R*-factors based on *F*^2^ are statistically about twice as large as those based on *F*, and *R*- factors based on ALL data will be even larger.

### Fractional atomic coordinates and isotropic or equivalent isotropic displacement parameters (Å^2^)

**Table:**

	*x*	*y*	*z*	*U*_iso_*/*U*_eq_	
N1	0.7041 (7)	0.41520 (18)	0.10705 (18)	0.0222 (9)	
N2	0.8001 (6)	0.29462 (17)	0.14467 (17)	0.0196 (9)	
N3	1.5730 (8)	0.3343 (2)	−0.1704 (2)	0.0366 (11)	
N4	0.1768 (8)	−0.0990 (2)	0.05749 (19)	0.0328 (10)	
C1	0.5702 (8)	0.3997 (2)	0.1633 (2)	0.0205 (10)	
C2	0.3933 (8)	0.4456 (2)	0.1943 (2)	0.0250 (11)	
H2A	0.3510	0.4953	0.1787	0.030*	
C3	0.2841 (8)	0.4148 (2)	0.2487 (2)	0.0271 (11)	
H3A	0.1639	0.4441	0.2697	0.033*	
C4	0.3485 (8)	0.3408 (2)	0.2733 (2)	0.0272 (11)	
H4A	0.2719	0.3223	0.3105	0.033*	
C5	0.5240 (8)	0.2944 (2)	0.2434 (2)	0.0240 (11)	
H5A	0.5681	0.2451	0.2597	0.029*	
C6	0.6310 (8)	0.3255 (2)	0.1876 (2)	0.0197 (10)	
C7	0.8349 (8)	0.3516 (2)	0.0971 (2)	0.0196 (10)	
C8	0.9937 (8)	0.3425 (2)	0.0400 (2)	0.0183 (10)	
C9	1.0085 (8)	0.2747 (2)	0.0032 (2)	0.0206 (11)	
H9A	0.9193	0.2310	0.0153	0.025*	
C10	1.1554 (8)	0.2718 (2)	−0.0515 (2)	0.0248 (11)	
H10A	1.1656	0.2262	−0.0758	0.030*	
C11	1.2870 (8)	0.3372 (2)	−0.0697 (2)	0.0207 (11)	
C12	1.2703 (8)	0.4059 (2)	−0.0344 (2)	0.0228 (11)	
H12A	1.3561	0.4499	−0.0473	0.027*	
C13	1.1248 (8)	0.4079 (2)	0.0200 (2)	0.0229 (11)	
H13A	1.1135	0.4538	0.0439	0.027*	
C14	0.9363 (8)	0.2212 (2)	0.1570 (2)	0.0221 (11)	
H14A	0.9987	0.2167	0.2057	0.026*	
H14B	1.0918	0.2209	0.1329	0.026*	
C15	0.7667 (8)	0.1513 (2)	0.1346 (2)	0.0193 (10)	
C16	0.8439 (8)	0.0795 (2)	0.1615 (2)	0.0232 (11)	
H16A	0.9962	0.0751	0.1935	0.028*	
C17	0.6958 (8)	0.0145 (2)	0.1410 (2)	0.0256 (11)	
H17A	0.7492	−0.0336	0.1589	0.031*	
C18	0.4669 (8)	0.0212 (2)	0.0936 (2)	0.0217 (11)	
C19	0.3883 (8)	0.0931 (2)	0.0667 (2)	0.0228 (11)	
H19A	0.2349	0.0978	0.0351	0.027*	
C20	0.5388 (8)	0.1572 (2)	0.0871 (2)	0.0208 (10)	
H20A	0.4869	0.2052	0.0687	0.025*	
C21	1.4437 (9)	0.3350 (2)	−0.1261 (3)	0.0281 (12)	
C22	0.3071 (9)	−0.0458 (2)	0.0734 (2)	0.0256 (11)	

### Atomic displacement parameters (Å^2^)

**Table:**

	*U*^11^	*U*^22^	*U*^33^	*U*^12^	*U*^13^	*U*^23^
N1	0.028 (2)	0.016 (2)	0.023 (2)	0.0017 (16)	0.0025 (19)	−0.0006 (16)
N2	0.026 (2)	0.0127 (19)	0.019 (2)	0.0003 (15)	0.0024 (18)	−0.0026 (16)
N3	0.043 (3)	0.031 (2)	0.036 (3)	−0.0030 (19)	0.006 (2)	−0.001 (2)
N4	0.045 (3)	0.027 (2)	0.027 (2)	−0.006 (2)	0.006 (2)	−0.0006 (18)
C1	0.026 (2)	0.016 (3)	0.019 (3)	−0.0022 (19)	0.003 (2)	−0.003 (2)
C2	0.029 (3)	0.015 (2)	0.030 (3)	0.003 (2)	0.000 (2)	−0.006 (2)
C3	0.025 (2)	0.031 (3)	0.027 (3)	−0.004 (2)	0.007 (2)	−0.006 (2)
C4	0.032 (3)	0.026 (3)	0.026 (3)	−0.008 (2)	0.008 (2)	−0.006 (2)
C5	0.028 (3)	0.019 (2)	0.024 (3)	−0.003 (2)	−0.002 (2)	−0.003 (2)
C6	0.024 (2)	0.016 (2)	0.018 (3)	−0.0035 (19)	0.001 (2)	−0.005 (2)
C7	0.021 (2)	0.017 (2)	0.019 (3)	−0.0054 (19)	−0.005 (2)	0.000 (2)
C8	0.021 (2)	0.015 (2)	0.019 (3)	0.0019 (18)	0.001 (2)	0.003 (2)
C9	0.023 (2)	0.016 (3)	0.023 (3)	−0.0008 (18)	0.003 (2)	0.004 (2)
C10	0.032 (3)	0.022 (3)	0.019 (3)	0.002 (2)	−0.001 (2)	−0.001 (2)
C11	0.022 (2)	0.021 (3)	0.020 (3)	0.0000 (19)	0.004 (2)	0.005 (2)
C12	0.031 (3)	0.017 (3)	0.021 (3)	−0.0037 (19)	0.004 (2)	−0.002 (2)
C13	0.031 (3)	0.015 (2)	0.022 (3)	0.002 (2)	0.002 (2)	−0.002 (2)
C14	0.023 (2)	0.021 (2)	0.023 (3)	0.0009 (19)	0.003 (2)	0.0011 (19)
C15	0.023 (3)	0.016 (2)	0.019 (3)	0.0013 (18)	0.005 (2)	−0.0030 (19)
C16	0.026 (2)	0.020 (2)	0.024 (3)	−0.001 (2)	0.004 (2)	0.001 (2)
C17	0.026 (3)	0.019 (2)	0.032 (3)	0.003 (2)	0.004 (3)	0.001 (2)
C18	0.026 (3)	0.017 (2)	0.024 (3)	−0.0035 (19)	0.010 (2)	−0.004 (2)
C19	0.021 (2)	0.024 (3)	0.022 (3)	0.000 (2)	0.001 (2)	0.001 (2)
C20	0.027 (3)	0.015 (2)	0.021 (3)	0.0006 (19)	0.004 (2)	−0.0002 (19)
C21	0.035 (3)	0.019 (3)	0.030 (3)	−0.003 (2)	0.003 (3)	0.000 (2)
C22	0.033 (3)	0.023 (3)	0.022 (3)	−0.001 (2)	0.007 (2)	0.002 (2)

### Geometric parameters (Å, °)

**Table:**

N1—C7	1.314 (5)	C10—C11	1.385 (5)
N1—C1	1.397 (5)	C10—H10A	0.9300
N2—C6	1.385 (5)	C11—C12	1.387 (5)
N2—C7	1.387 (5)	C11—C21	1.446 (6)
N2—C14	1.453 (5)	C12—C13	1.376 (5)
N3—C21	1.155 (5)	C12—H12A	0.9300
N4—C22	1.152 (5)	C13—H13A	0.9300
C1—C6	1.391 (5)	C14—C15	1.515 (5)
C1—C2	1.397 (5)	C14—H14A	0.9700
C2—C3	1.375 (6)	C14—H14B	0.9700
C2—H2A	0.9300	C15—C20	1.386 (5)
C3—C4	1.393 (6)	C15—C16	1.388 (5)
C3—H3A	0.9300	C16—C17	1.384 (5)
C4—C5	1.385 (6)	C16—H16A	0.9300
C4—H4A	0.9300	C17—C18	1.390 (5)
C5—C6	1.394 (6)	C17—H17A	0.9300
C5—H5A	0.9300	C18—C19	1.391 (5)
C7—C8	1.472 (6)	C18—C22	1.440 (6)
C8—C9	1.387 (5)	C19—C20	1.377 (5)
C8—C13	1.397 (5)	C19—H19A	0.9300
C9—C10	1.385 (5)	C20—H20A	0.9300
C9—H9A	0.9300		
			
C7—N1—C1	105.2 (3)	C10—C11—C21	120.6 (4)
C6—N2—C7	106.1 (3)	C12—C11—C21	118.8 (4)
C6—N2—C14	123.7 (3)	C13—C12—C11	119.0 (4)
C7—N2—C14	129.6 (3)	C13—C12—H12A	120.5
C6—C1—C2	120.2 (4)	C11—C12—H12A	120.5
C6—C1—N1	109.9 (4)	C12—C13—C8	121.3 (4)
C2—C1—N1	129.8 (4)	C12—C13—H13A	119.3
C3—C2—C1	117.6 (4)	C8—C13—H13A	119.3
C3—C2—H2A	121.2	N2—C14—C15	114.6 (3)
C1—C2—H2A	121.2	N2—C14—H14A	108.6
C2—C3—C4	121.8 (4)	C15—C14—H14A	108.6
C2—C3—H3A	119.1	N2—C14—H14B	108.6
C4—C3—H3A	119.1	C15—C14—H14B	108.6
C5—C4—C3	121.5 (4)	H14A—C14—H14B	107.6
C5—C4—H4A	119.3	C20—C15—C16	119.3 (4)
C3—C4—H4A	119.3	C20—C15—C14	121.6 (3)
C4—C5—C6	116.4 (4)	C16—C15—C14	119.2 (4)
C4—C5—H5A	121.8	C17—C16—C15	120.4 (4)
C6—C5—H5A	121.8	C17—C16—H16A	119.8
N2—C6—C1	106.0 (4)	C15—C16—H16A	119.8
N2—C6—C5	131.6 (4)	C16—C17—C18	119.7 (4)
C1—C6—C5	122.4 (4)	C16—C17—H17A	120.1
N1—C7—N2	112.7 (4)	C18—C17—H17A	120.1
N1—C7—C8	122.6 (4)	C17—C18—C19	120.0 (4)
N2—C7—C8	124.6 (4)	C17—C18—C22	120.2 (4)
C9—C8—C13	118.8 (4)	C19—C18—C22	119.8 (4)
C9—C8—C7	124.0 (4)	C20—C19—C18	119.6 (4)
C13—C8—C7	117.1 (4)	C20—C19—H19A	120.2
C10—C9—C8	120.4 (4)	C18—C19—H19A	120.2
C10—C9—H9A	119.8	C19—C20—C15	120.9 (4)
C8—C9—H9A	119.8	C19—C20—H20A	119.6
C9—C10—C11	119.8 (4)	C15—C20—H20A	119.6
C9—C10—H10A	120.1	N3—C21—C11	178.5 (5)
C11—C10—H10A	120.1	N4—C22—C18	179.2 (5)
C10—C11—C12	120.7 (4)		
			
C7—N1—C1—C6	1.5 (4)	N2—C7—C8—C13	−147.4 (4)
C7—N1—C1—C2	−177.1 (4)	C13—C8—C9—C10	1.3 (6)
C6—C1—C2—C3	−0.1 (6)	C7—C8—C9—C10	177.7 (4)
N1—C1—C2—C3	178.4 (4)	C8—C9—C10—C11	−0.3 (6)
C1—C2—C3—C4	0.9 (6)	C9—C10—C11—C12	−0.9 (6)
C2—C3—C4—C5	−0.7 (6)	C9—C10—C11—C21	179.3 (4)
C3—C4—C5—C6	−0.3 (6)	C10—C11—C12—C13	1.2 (6)
C7—N2—C6—C1	0.4 (4)	C21—C11—C12—C13	−179.0 (4)
C14—N2—C6—C1	172.4 (3)	C11—C12—C13—C8	−0.2 (6)
C7—N2—C6—C5	178.7 (4)	C9—C8—C13—C12	−1.0 (6)
C14—N2—C6—C5	−9.3 (6)	C7—C8—C13—C12	−177.7 (4)
C2—C1—C6—N2	177.5 (3)	C6—N2—C14—C15	81.2 (5)
N1—C1—C6—N2	−1.2 (4)	C7—N2—C14—C15	−108.9 (4)
C2—C1—C6—C5	−1.0 (6)	N2—C14—C15—C20	20.2 (5)
N1—C1—C6—C5	−179.7 (4)	N2—C14—C15—C16	−160.9 (4)
C4—C5—C6—N2	−177.0 (4)	C20—C15—C16—C17	0.2 (6)
C4—C5—C6—C1	1.1 (6)	C14—C15—C16—C17	−178.7 (4)
C1—N1—C7—N2	−1.3 (4)	C15—C16—C17—C18	−0.6 (6)
C1—N1—C7—C8	177.1 (3)	C16—C17—C18—C19	0.4 (6)
C6—N2—C7—N1	0.5 (4)	C16—C17—C18—C22	−178.2 (4)
C14—N2—C7—N1	−170.8 (4)	C17—C18—C19—C20	0.2 (6)
C6—N2—C7—C8	−177.8 (4)	C22—C18—C19—C20	178.7 (4)
C14—N2—C7—C8	10.9 (6)	C18—C19—C20—C15	−0.6 (6)
N1—C7—C8—C9	−142.1 (4)	C16—C15—C20—C19	0.3 (6)
N2—C7—C8—C9	36.1 (6)	C14—C15—C20—C19	179.3 (4)
N1—C7—C8—C13	34.4 (5)		

### Hydrogen-bond geometry (Å, °)

**Table:**

*D*—H···*A*	*D*—H	H···*A*	*D*···*A*	*D*—H···*A*
C12—H12A···N1^i^	0.93	2.62	3.425 (5)	146
C19—H19A···N4^ii^	0.93	2.57	3.502 (6)	175
C9—H9A···N4^iii^	0.93	2.71	3.361 (5)	128
C14—H14A···N3^iv^	0.97	2.57	3.502 (5)	162

Symmetry codes: (i) −*x*+2, −*y*+1, −*z*; (ii) −*x*, −*y*, −*z*; (iii) −*x*+1, −*y*, −*z*; (iv) *x*−1/2, −*y*+1/2, *z*+1/2.
